# Treatment of Fracture of the Jaw, with Critical Remarks

**Published:** 1880-06

**Authors:** Thomas Brian Gunning

**Affiliations:** New York


ARTICLE V. PART II.

### TREATMENT OF FRACTURE OF THE JAW, WITH CRITICAL REMARKS.

BY THOMAS BRIAN GUNNING, D. D. S., NEW YORK.

Diagnosis of fracture of the jaw will be easier after recapitulation of the muscular action in connection with the leverage afforded by the jaw, and also the base of the skull through which the muscular power is made effective.

The head, as generally seen, appears so firmly supported by the neck, that notwithstanding the extent of its movements, and the ease with which they are effected, it is difficult to realize the great flexibility of the cervical spine; unless this is done, however, it is impossible to understand the mechanical relations existing between the jaw, head, neck and body.

Although this flexibility of the cervical spine can not be precisely estimated, a sufficiently correct idea may be attained by letting the head *fall* in different directions and making allowance for the surrounding muscles, fasciæ, blood vessels, nerves, &c. It will then be felt that the cervical vertebræ and their ligaments could not protect the spinal marrow and support the head, even with the aid of the muscles which cleave to them, except for the assistance of the muscles attached to parts more removed from the centres upon which the head rocks and turns. These muscles, however, are not attached to the spine but to the skull, it follows that when they put the great weight of the head in motion, the spine goes with it, keeping the head extended in every direction and supporting it on the atlas when the body is upright.

The head, therefore, moves the cervical vertebræ, and its leverage must be considered.

Of the whole distance between the origins of the genio-hyoid and the digastrics inside the jaw to those of the *trapezii* on the occipital bone, or the distance between the most anterior muscles which act upon the point of the jaw, and of the most posterior which act upon the base of the skull; from $\frac{4}{7}$ to $\frac{2}{3}$ is in front of the centre, upon which the head rocks. The condyles of the lower jaw are, with the teeth closed on a line a little in front of this centre, and each condyle is separated so far from its respective coronoid process that they form of each ramus, which projects forward in front of each ear, a horizontal lever half as long as the distance between the centre upon which the head rocks and the inside of the chin. Thus the temporal muscle, which is inserted into the anterior end of this lever, in connection with the *external pterygoid*, inserted into its posterior termination can hold the jaw so firmly in any position, that it is virtually, when so held a part of the skull or in other words it is the great lever through which the head is controlled by means of the muscles which pass to the hyoid bone.

The hyoid bone gives insertion to the *genio-hyo-glossus*, and the *genio-hyoid*, which arise on the inside of the jaw near the symphysis, and to the *mylo-hyoid* which descends from around the side of the body of the jaw; while the *sterno-hyoid* which passes up from the breast bone completes the line of muscular control between the front of the trunk and the point of the lower jaw. Below the *mylo-hyoids* (or floor of the mouth,) the anterior *digastrics* also *arise* around the inside of the front of the jaw, but in going back diverge to their tendinous junction with the posterior bellies which come down from their origins on the head inside of the mastoid processes; and under the *aponeurotic* loops which hold the tendons of the digastrics to the *hyoid* bone the *homo-hyoid* muscles are inserted into its lower border; each muscle having passed on its own side of the neck from its origin on the scapula, &c., and after crossing the shoulder and by its tendon, which separates its two muscular bellies, passing through a loop of the deep cervical fascia which attaches it loosely to the cartilage of the first rib, ascends almost vertically to the hyoid bone. Thus the lower jaw (termed a floating bone from its facile mobility,) is held by these two pairs of double bellied muscles by means of the hyoid bone, in relation both to the back part of the trunk and to the least movable part of the head; while the muscles which pass from the sides and base of the skull to the *coronoid* processes and condyles of the jaw, control its movements so completely that the body of the jaw affords a base from which the anterior bellies of the digastrics antagonize or act in concert with their posterior bellies and with the *omo-hyoid* and other muscles. When the body is back out of the perpendicular, and even when the head only is over back, it is brought into balance through the great leverage of the jaw, by the muscles which pass from the sternum and shoulders. These muscles act upon the body of the jaw, the horizontal lever, which is on an average twice as long in front of the centre of the head's support as the vertical lever of the ramus is below it. The longer lever brings the head up and forward until it comes under the control of the vertical lever of the ramus; the head being controlled by the mixed leverage brought into use by going from one to the other and this whether the head is going back or coming forward.

The use of the jaw in controlling the head, or when acting as a part of the skull is now demonstrated.

The head and jaw will now be considered in the functions connected with food and speech, &c., in which, while the head is still, the jaw is active.

With the body upright and the head at rest upon the atlas, more than half the weight of the head is forward of its centre of support, and the anterior recti muscles, although so near the centre, effectually antagonize the complexi and other muscles behind the condyles. These muscles all act vertically upon the horizontal lever of the skull and control it in its *antero-posterior* directions. The lateral movements of the head not being considered in this paper.

It has been shown that in movements of the head its muscles control the jaw by means of the rami, and of course, this is also done in the special functions now under consideration.

In mastication, the muscular action between the head and jaw is increased in proportion to the resisting quality of the food, yet little or no movement of the head results, although the temporal muscles are active, nor even when the great force of the *masseters* is engaged in crushing the hardest substances, if the jaw is allowed to go down *unrestricted.* The supposed upward movement of the head in mastication has been speculated upon by many investigators, Monroe, Pringle, Bèrard, Ferrien, Hunter; but without finding a solution of the question. Dr. Austin Flint, Jr., says: "The movement of the head, however, does not ordinarily require any powerful muscular action, and is probably the result of the contraction of muscles too deeply situated to be explored experimentally."* In that year, however, my discovery that the *external pterygoid* was the depressor of the jaw was published; † and the support of the chin to demonstrate the action of the external pterygoid, solved the difficulty; the head moves up in mastication only when the jaw is prevented from going down, and this whether by a cravat or by the mantel-piece. The muscles brought into use in mastication including even the *internal pterygoid*, arise on the skull, in front of the atlas and move the jaw only. Deglutition brings other muscles into use, whose action is more complicated.

In this function the jaw is so held that its teeth nearly touch the upper ones, and in order that the tongue may make air-tight contact with the roof of the mouth, there is upward movement of the hyoid bone and its attachments; this is effected by the palate and other muscles which depend on the skull, not by those which arise on the jaw. If the mouth is opened wide and the tongue drawn up to swallow, even the point of the *thyroid, cartilage* will be above the lower border of the jaw; the *external pterygoid* holding the latter down and thus demonstrating its power to open the mouth. The muscles which arise inside the front of the jaw antagonize the constrictors of the pharynx and those muscles which arise on the styloid process of the temporal bone and in the digastric notch. These all act in a horizontal direction and most strongly when the hyoid bone is drawn up within the body of the jaw. In respect to this horizontal action of the muscles upon the skull, it may be concluded that its influence is very slight, and is such as counteracts the vertical action, for the inferior and middle constrictors arise on the spine below the axis, while the styloid processes which are virtually on a line with the centre upon which the head rocks, extend downward, so that the effect of the styloid muscles, as with the constrictors, if any, must be to throw the head upward, or rather to neutralize the downward action of the palate muscles. With mastication there is more or less suction to get

*The Physiology of Man, vol. 2, p. 148, D. Appleton & Co., N. Y., 1867.

†New York Medical Journal, vol. vi, p. 211.

the food into the mouth, between the teeth and under control of the pharyngeal muscles. This is effected by the *digastrics* and *omohyoids* whose action on the jaw is somewhat like that which it meets as the head passes from the control of the horizontal lever of the body to the vertical lever of the ramus. The great weight of the head, however, is moved but little in the transient movements of suction, while the action in the floor of the mouth affects the jaw considerably.

In speech the vocal utterance may be said to affect the head similarly to the upward movements of the hyoid bone in deglutition and while the tongue in articulating the lingual consonants, is unaided by special movement in the jaw, during any particular vowel-sound of one continued pitch of tone, yet in changing the vowel the jaw alters its position, and this so much in tones of high pitch that the mouth is opened very wide, but this does not act on the head more forcibly than deglutition, nor does it require stronger action of the muscles behind the atlas.

These remarks are sufficiently explicit; they suggest the effect of the muscular action, rather than estimate it precisely; this last being impossible. For in addition to the difference between the muscles of different persons and of the same person under the changes of age and condition, the form and size of the jaw and of the skull vary so much that the most careful estimate of their leverage can give but approximate ideas of any special case.

It has, however, been demonstrated that the *sterno-mastoid* muscle does not flex the head upon the atlas and neck, but that this is done by means of the lower jaw.

It was necessary to make the foregoing views clear, even at the expense of so much attention, for my paper in the New York Medical Journal in 1867, showed that the *external pterygoid* was the depressor of the jaw and that the *sterno-mastoid* muscle does not rock the head upon the atlas.

It also maintained that the *sterno-mastoids* do not usually rotate the head; this being necessary, although the supposition that they did was declared by Dr. John Barclay to be a "vulgar notion," nearly sixty years earlier. Again,

Professor Harrison Allen, M. D., in his Studies on the Facial Regin,* says:

*J. B. Lippincott & Co., Philadelphia, 1875.

"The massive internal pterygoid and the masseter act together in directly raising the lower jaw. But before it can do this, the condyloid process, which has been tilted out of the glenoid fossa, must be replaced. This is done by the external pterygoid; the larger slip operating on the jaw, the lesser one adjusting the capsule and the inter-articular disk." This absurd statement, for to do so the external pterygoid must push the condyle and cartilage back, was first published in November, 1873,* so that ample time had been given for criticism and correction to have prevented it from being again sent out to mislead. Yet it was re-published in the eighth year after I had demonstrated that the external pterygoid is the depressor of the lower jaw.

In the same year, 1875, "The Leverage of the Lower Human Jaw, by John Gorham, F. R. C. S., in Medical Times and Gazette," was re-published in Philadelphia.† The author elaborated his views very scientifically; he speaks of the jaw as a lever of the third kind, because the muscular power is applied between the condyle and the part which holds the teeth, the weight of which and their positions being carefully laid down, but no consideration is given to the other parts attached to the body of the jaw, although the effect of their action in function is attributed to it.

It may be admitted that, although the condyle moves back, it is still the fulcrum upon which the weight of the teeth and jaw, &c., are carried up, as they go back with it. The paper, however, as a whole, is radically defective and misleads. The frequent reference to the leverage of the jaw in its upward movement without remark upon the backward and forward movement of the condyle to shut and open the teeth, leaves no place to hope that the author had any idea that the jaw was depressed as a lever of the first kind, the power being applied at the condyle the end of the rod, the fulcrum being between the power and the teeth, the weight; and this whether the fulcrum is held to be at the insertion of the stylo-maxillary ligament on the angle or at that of the internal lateral ligament, around the inferior dental foramen. This omission effectually shuts out all idea that the external pterygoid depresses the jaw. Further, so much trouble to estimate the weight of the teeth, leaves no reason to suppose that the jaw is the lever by which the head is controlled. Mr. Gorham's paper,

*Dental Cosmos, Vol. XV, p. 568.

†Dental Cosmos, Vol. XVII, pp. 212, 267.

however, is a pleasant assurance of his industry and unusual ability for scientific investigation. It also deepens the conviction that my own remarks upon the muscular action of these parts will be of service to many. Concluding summary. The muscles which give form to the neck and support and move the head, and through it the cervical spine, are so arranged as to leave the fronts of the vertebræ free for the tongue, hyoid-bone, larynx and trachea, with their special muscles. These organs being attached to and in part surrounded by the body of the lower jaw, from which also many of their muscles act to move them in their functions—deglutition and vocalization, &c., while its own movements are made to co-operate in these functions and in mastication and articulation by muscles which arise on the head and are inserted into the superior terminations of the rami. In these functions the front of the jaw is a base from which the muscles act to antagonize those which arise on the skull and the back of the pharynx, while in all motions of the head the jaw is the great lever through which the muscles give anterior support to and exert control of the head. The radical importance of the lower jaw and of the integrity of its leverage is now demonstrated, and due allowance can be given in judging of and treating its fractures.

---

### DIAGNOSIS OF FRACTURE OF THE JAW.

That this, the foundation of correct treatment, may be placed in its most simple features before the reader; little will be said of gun-shot fractures as they are often so anomalous as to confuse rather than assist the student to comprehend the principles which lead to correct views regarding fracture of the lower jaw.

This accords with my presentation of the Treatment of Fracture of the Jaw by "Interdental Splints," in 1866, when severe injuries, brought under my observation during the war, were left out. The EIGHT cases then taken to show the use of the *splints*, were selected to give a clear and full view of all which I considered essential to hold any broken jaw in place; several other cases being referred to. The experience of fourteen years which have since passed justifies me in republishing these cases, together with others now selected to assist in giving clear ideas upon the diagnosis of fracture of the jaw.

The jaw may break in the symphysis, in any part of the body, in one or both angles, necks of its condyles, or in its coronoid processes, and in several different places at the same time. Enumeration of the position of these fractures, or rather the proportion which fractures in a particular part bear to those in any or to all other parts gives but little assistance. Age, however, is suggestive, for in very young children the symphysis or the body gives way easily compared with the parts back of the teeth; the symphysis not being ossified until some time in the second year, and later, the body in its upper, the alveolar border is excavated for the germs of the permanent teeth as well as the teeth of the infant set, which last at three years of age are fully developed and standing in the mouth. As this temporary set gives place to the longer teeth of the adult, the bone below the alveolar process grows deeper and stronger and its obtuse angle gradually becomes more vertical by which the neck of the condyle is more exposed to fracture from blows upon the front of the jaw. But, notwithstanding the comparatively greater risk to the neck of the condyle in adult life, its fracture is very rare compared with those in the body of the jaw. The exposure of mature life is of course followed by a large increase in fractures of the jaw, and this although the body of the jaw, is so fully developed both in the true bone and, the deep sockets of the teeth.

These sockets, however, seem to offer a starting place for fracture, the greater proportion being found in the alveolar when holding teeth. The singular immunity of the lower jaw, when without teeth is shown in the fact that Malgaigne says: *"Duverney has suggested that the jaw might be broken when completely destitute of teeth. This case seems never to have presented itself." My own observations reach back to 1840, but no case of a lower jaw fractured when destitute of teeth, has come to my knowledge, although I have seen the jaw so absorbed under the use of very heavy lower plates of artificial teeth that the body was only a quarter of an inch deep in the parts where the first permanent molars had stood. In April 1879, however, a farmer 70 years old fell down stairs and broke his jaw in the socket of the right canine, which was knocked out, and the mouth left without a tooth or root in it.† Therefore, M. Malgaignes' exception has presented itself as he referred to treatment; but although no jaw is yet known to have broken that was destitute of teeth; there should

*J. F. Malgaigne, TREATISE ON FRACTURES. Translated from the French by John H. Packard, M. D., p. 321. J. B. Lippincott & Co., Phil. 1859.

†The case was successfully treated by Dr. J. A. Bishop, and is reported in Johnstons Dental Miscellany, VOL. VII, p. 63.

be no doubt that lower jaws without teeth will break in the body much easier than in any other part.

The fact that fractures are met with precisely in the symphysis, suggests distrust as to the completion of ossification of this part in some jaws; with its two halves firmly united it seems much more likely that the bone would break in the socket of a tooth.

(*TO BE CONTINUED.*)

